# Heat Shock Protein 27 (HSP27) as a Potential Prognostic Marker: Immunohistochemical Analysis of Oral Epithelial Dysplasia and Oral Squamous Cell Carcinoma

**DOI:** 10.7759/cureus.33020

**Published:** 2022-12-27

**Authors:** Karthikeyan Ramalingam, Gurveen Chawla, Abhiney Puri, Murugesan Krishnan, Tania Aneja, Kaajal Gill

**Affiliations:** 1 Oral Pathology and Microbiology, Saveetha Dental College and Hospitals, Saveetha Institute of Medical and Technical Sciences, Chennai, IND; 2 Oral Pathology, Surendera Dental College and Research Institute, Sriganganagar, IND; 3 Oral Pathology, Himachal Institute of Dental Sciences, Paonta Sahib, IND; 4 Oral and Maxillofacial Surgery, Saveetha Dental College and Hospitals, Saveetha Institute of Medical and Technical Sciences, Chennai, IND; 5 Dentistry, Department of Medical Health and Family Welfare, Uttarakhand, IND

**Keywords:** prognosis, dysplasia, oral cancer, hsp27, heat shock proteins

## Abstract

Aim: To evaluate and correlate the expression of heat shock protein 27 (HSP27) in oral epithelial dysplasia (ED) and oral squamous cell carcinoma (OSCC).

Materials and methods: This immunohistochemical study of HSP27 expression was performed on 45 samples retrieved from the departmental archives. It included 15 cases of oral ED, 15 cases of OSCC and 15 cases of epithelial hyperplasia (EH). The staining intensity and distribution were scored. The expression was compared between the study groups. Kruskal-Wallis Test, Mann-Whitney U Test and Chi-square tests were performed for statistical analysis using SPSS v21.0 (IBM Corp., Armonk, NY, USA).

Results: There was a statistically significant difference in HSP27 staining parameters between EH, oral ED and OSCC. There was no significant difference between oral ED and OSCC.

Conclusion: HSP27 expression shows enhanced expression in oral ED and OSCC. Its expression should be investigated using larger sample sizes with clinico-pathological correlation to prove its efficiency as a prognostic marker. It will help us in defining treatment modalities so that mortality and morbidity associated with OSCC could be reduced.

## Introduction

In the global scenario, cancer of the head and neck is ranked third among common malignancies noted in developing countries. Squamous cell carcinoma is the most frequent with a 46% survival rate [1]. Eighty-six to 95% of such head and neck malignancies arise from the surface epithelium. Oral squamous cell carcinoma (OSCC) can involve any part of the oral cavity and oropharynx but predominantly involves the tongue. A multitude of risk factors and genetic determinants play a critical role in its pathogenesis [2,3].

Most patients with OSCC report at advanced stages that require radical surgery, higher doses of radiotherapy and chemotherapy that severely impacts speech and swallowing and compromises quality of life. Though multiple tumor markers have potential, an OSCC screening tool with an accurate diagnostic and prognostic marker is still a mirage [4].

Oral potentially malignant disorders have varied clinical presentations and it is difficult to predict the evolution of malignant change. Prognosis could be determined by histopathology and expression of molecular markers. The heat shock protein (HSP) family promotes growth and survival by enhancing stress resistance in tumor cells. HSPs also increase tumor immunity by stimulation of innate immune responses [1].

These proteins, primarily the HSP70 family, HSP90 family, and HSP27, are essential for normal homeostasis of the body. HSPs prevent protein coagulation and maintain multi-protein complexes in stress-induced states. HSPs regulate refolding of damaged proteins and destruction of misfolded/short-lived proteins. HSPs exert their anti-apoptotic effect by interacting with Bax, Cytochrome C and DAXX [5]. The overabundance of HSPs in stressed and transformed cells could favor anti-apoptotic activity [6].

Elevated expression of HSP27 either individually or in combination has been widely reported in cancers of breast, uterus and kidney, osteosarcoma and leukemias [6]. HSPs are produced transiently after cellular stressor events like heat shock or chemical and oxidative stress. HSPs are often overexpressed in tumor cells and reduce the response to chemotherapy with their anti-apoptotic capabilities [7,8]. HSP expression is labeled as a marker of the malignant potential of oral epithelial lesions [9].

The interesting possibility is that elevated HSP levels in tumor cells could impart a selective pro-survival edge and aid in carcinogenesis [10-12].

## Materials and methods

This retrospective study with a total of 45 specimens of formalin-fixed paraffin-embedded blocks of previously diagnosed patients were taken from the department archives. Of these 45 specimens, three groups were formed, Group 1: Oral Squamous Cell Carcinoma (OSCC), Group 2: Oral Epithelial Dysplasia (ED) and Group 3: Epithelial Hyperplasia (EH) with 15 samples for each group. Sections of each tissue (3-4 micrometers thick) were prepared with a Leica RM2245 microtome (Leica, Wetzlar, Germany).

One section was taken on a normal slide for routine hematoxylin and eosin stain as described in Bancroft [13]. Another section taken on a Poly-L-Lysine coated slide was stained for immunohistochemistry of HSP27, as per manufacturer's instructions (Biogenex Inc, Houston, TX, USA). Harris Hematoxylin was used as the counterstain. Appropriate positive and negative controls were utilized for every batch.

Photomicrographs were captured with an LX400 Labomed Inc. (Los Angeles, CA, USA) microscope and assessed for staining characteristics. Histopathological diagnosis of stained slides of OSCC, ED and EH were evaluated by three independent Oral Pathologists for representative histopathology, according to the World Health Organization classification. 

The staining intensity was graded on a scale of 0-4 and the mean percentage of positive cells in 5 high power fields showing maximum staining of HSP27 was used to grade 1-4. Staining Percentage was given as Score 1 for 0-25% stained cells, Score 2 for 25-50% stained cells, Score 3 for 50-75% stained cells and Score 4 for 75-100% stained cells [1]. Staining Intensity was given as Score 0 for No detectable immunoreactivity, Score 1 for Very low staining, Score 2 for Low staining, Score 3 for Moderate staining and Score 4 for High staining [1].

Staining index (SI) was calculated by multiplying staining percentage and staining intensity. Mean values of the staining index along with staining intensity (SIN) and percentage of stained cells (PSC) by three observers were tabulated and graphically plotted [1].

Difference in the staining index of epithelial cells between groups was analyzed with Kruskal-Wallis Test, Mann-Whitney U Test and Chi-square tests performed using SPSS v21.0 (IBM Corp., Armonk, NY, USA). p ≤ 0.05 was considered statistically significant.

Saveetha Dental College Institutional Human Ethics Committee issued approval IHEC/SDC/PhD/OPATH-2212/22/001.

## Results

In normal epithelium the staining was present only in the basal cells. Among 45 cases of in the present study, 15 cases were oral ED (with five cases each of mild dysplasia, moderate dysplasia and severe dysplasia), 15 cases were OSCC (with five cases each of well differentiated SCC, moderately differentiated SCC and poorly differentiated SCC) and 15 had EH (Table 1).

**Table 1:** Distribution of samples among three study groups EH - Epithelial Hyperplasia, ED - Oral Epithelial Dysplasia, OSCC - Oral Squamous Cell Carcinoma, MDSCC - Moderately Differentiated Squamous Cell Carcinoma, WDSCC - Well differentiated Squamous Cell Carcinoma, PDSCC - Poorly Differentiated Squamous Cell Carcinoma

Groups	Description		Number Of Cases (N)	Total
Group 3	EH		15	15
Group 2	ED	Mild	5	15
		Moderate	5	
		Severe	5	
Group 1	OSCC	MDSCC	5	15
		WDSCC	5	
		PDSCC	5	
				45

Mean staining index was highest in OSCC (6.07) followed by ED (2.40) and least in EH (1) (Table 2).

**Table 2:** Results of Kruskal-Wallis Test for mean staining index (SI) of epithelial cells for HSP27 in hyperplastic tissue, dysplasia and oral squamous cell carcinoma EH- Epithelial Hyperplasia, ED - Oral Epithelial Dysplasia, OSCC - Oral Squamous Cell Carcinoma

Groups	N	Mean SI	Mean Rank	p value	Chi-Square	df
EH	15	1	14.83	0.003**	11.394	2
ED	15	2.4	23.47			
OSCC	15	6.07	30.7			
Total	45					
p value= 0.003** (significant)						

Mann-Whitney Test was performed between three groups. HSP27 expression was higher in ED when compared with EH with p value of 0.027. Comparison with EH and OSCC was also significant with p value of 0.003. But, the comparison between ED and OSCC was insignificant with p value of 0.062 (Table 3).

**Table 3:** Results of Mann-Whitney Test for mean staining index (SI) of epithelial cells for HSP27 in hyperplastic tissue, dysplasia and oral squamous cell carcinoma EH - Epithelial Hyperplasia, ED - Oral Epithelial Dysplasia, OSCC - Oral Squamous Cell Carcinoma

Groups	N	Mean SI	Mean Rank	Sum of Ranks	Mann-Whitney Test	W	p value
EH	15	1	12.07	181	61	181	0.027**
ED	15	2.4	18.93	284			
EH	15	1	10.77	161.5	41.5	161.5	0.003**
OSCC	15	6.07	20.23	303.5			
ED	15	2.4	12.53	188	68	188	0.062**
OSCC	15	6.07	18.47	277			
p value= 0.027** and 0.003** (significant), 0.062**(insignificant)							

Results of the staining index of HSP27 among three groups of dysplasia were seen to be more in mild dysplasia (3.2) than severe dysplasia (2.4) and was minimum in moderate dysplasia (1.6). It did not achieve statistical significance as p value was 0.309 (Table 4).

**Table 4:** Kruskal-Wallis Test of mean staining index (SI) among dysplastic subgroups MD - Mild Dysplasia, MOD - Moderate Dysplasia, SD - Severe Dysplasia

Groups	N	Mean SI	Mean Rank	p value	Chi-Square	Df
MD	5	3.2	10.4	0.309**	2.348	2
MOD	5	1.6	7			
SD	5	2.4	6.6			
Total	15					
p value= 0.309**(insignificant)						

Mann Whitney test for mean staining index of epithelial cells for HSP27 shows no significant differences between mild and severe dysplasia (p=0.220), moderate and severe dysplasia (p=0.737), mild and moderate dysplasia (p=0.167) (Table 5).

**Table 5:** Results of Mann-Whitney Test for mean staining index (SI) of epithelial cells for HSP27 in dysplastic subgroups MD - Mild Dysplasia, MOD - Moderate Dysplasia, SD - Severe Dysplasia

Groups	N	Mean SI	Mean Rank	Sum of Ranks	Mann-Whitney Test	W	p value
MD	5	3.2	6.8	34	6	21	0.167**
MOD	5	1.6	4.2	21			
MD	5	3.2	6.6	33	7	22	0.220**
SD	5	2.4	4.4	22			
MOD	5	1.6	5.8	29	11	26	0.737**
SD	5	2.4	5.2	26			
p value=0.167**, 0.220** and 0.737**(insignificant)							

Kruskal-Willis Test for the staining index of HSP27 among three groups of OSCC was more in MDSCC (8.2) than PDSCC (6.2) and was minimum in WDSCC (3.8) but did not achieve statistical significance (p=0.452) (Table 6).

**Table 6:** Results of Kruskal-Wallis test for mean staining index (SI) of epithelial cells for HSP27 in OSCC subgroups MDSCC - Moderately Differentiated Squamous Cell Carcinoma, WDSCC - Well differentiated Squamous Cell Carcinoma, PDSCC - Poorly Differentiated Squamous Cell Carcinoma

Groups	N	Mean SI	Mean Rank	p value	Chi-Square	Df
WDSCC	5	3.8	6.1	0.452**	1.588	2
MDSCC	5	8.2	9.6			
PDSCC	5	6.2	8.3			
Total	15					
p value= 0.452**						

Mann-Whitney test for the mean staining index of OSCC subgroups did not achieve statistical significance. Comparison between WDSCC and MDSCC (p=0.248), WDSCC and PDSCC (p=0.399), MDSCC and PDSCC (0.599) (Table 7).

**Table 7:** Results of Mann-Whitney test for mean staining index (SI) of epithelial cells for HSP27 in OSCC subgroups MDSCC - Moderately Differentiated Squamous Cell Carcinoma, WDSCC - Well differentiated Squamous Cell Carcinoma, PDSCC - Poorly Differentiated Squamous Cell Carcinoma

Groups	N	Mean SI	Mean Rank	Sum of Ranks	Mann-Whitney test	W	p value
WDSCC	5	3.8	4.4	22	7	22	0.248**
MDSCC	5	8.2	6.6	33			
WDSCC	5	3.8	4.7	23.5	8.5	23.5	0.399**
PDSCC	5	6.2	6.3	31.5			
MDSCC	5	8.2	6	30	10	25	0.599**
PDSCC	5	6.2	5	25			
p value= 0.248**, 0.399** and 0.599** (insignificant)							

## Discussion

Family of heat shock proteins (HSPs) can expedite growth and survival of tumor cells due to their cytoprotective properties. HSP27 has an anti-apoptotic role with its mitochondrial interplay and can promote cancer progression. Increase in free radicals production leads to enhanced HSP27 expression to protect the cells from oxidative damage. Tumor cells of OSCC show higher levels of free radicals and could be responsible for amplified HSP27 response [1,2]. Increase in anti-apoptotic nuclear factor kappa-light chain enhancer of activated B cells (NF-κB) activity through enhanced degradation of its main inhibitor, inhibitor of kappa B (I-κB) may contribute to the anti-apoptotic effect of HSP27 [4]. Research has revealed a robust correlation between OSCC and HSP27. Variations in HSP27 have been implied in carcinogenesis [11,12].

According to the studies conducted by Seoane et al. [14], Ito et al. [15], Leonardi et al. [16], Tekkesin et al. [3], and Lo et al. [12], the expression of HSP27 increases in dysplasia and OSCC when compared to its expression in normal and hyperplastic epithelium. Another role for HSP27, that of facilitating the activation of the ubiquitin-proteasome pathway [17]. Increased HSP27 expression in oral lichen planus when compared to normal epithelium was shown by Bramanti et al. [18] and Tyagi et al. [19].

In our study, epithelial cells in OSCC showed higher intensity than the ED, whereas HSP27 staining of epithelial cells was minimal in case of EH. Kruskall-Wallis test showed that there was statistically significant difference among the three study groups in the mean staining index of epithelial cells for HSP27. Furthermore, Mann Whitney test for mean staining index of epithelial cells for HSP27 showed significant differences in EH and ED (p=0.027), EH and OSCC (p=0.002) and insignificant differences between ED and OSCC (p=0.062). Consistent with the above studies, our study also had progressive increase in expression of HSP27 among hyperplastic, dysplastic and carcinomatous lesions.

When the expression level of HSP27 in our study was compared within grades of dysplasia, the results show insignificant decrease in expression from mild to moderate dysplasia and insignificant increase from moderate to severe dysplasia. It could be attributed to the smaller sample size in our study. Expression level of HSP27 decreases with the increase in grades of dysplasia as stated by Leonardi et al. [16]. The downregulation in dysplasia could indicate the defective cytoprotection against mutagenic environmental factors and thus augment the transformation of oral epithelial dysplasia into OSCCs.

Our results were consistent with these studies but the results were insignificant when all grades of OSCC were compared for the expression level of HSP27. Our study results show higher expression of HSP27 in MDSCC as compared to PDSCC. Nakajima et al. [20], Muzio et al. [21], Wang et al. [2] and Leonardi et al. [16] reported inverse relation of expression of HSP27 with increase in grades of OSCC and stated that expression of HSP27 decrease as the grades of OSCC increase from WDSCC to PDSCC.

Prognostic role of HSP27 in OSCC is still ambiguous. Some studies have reported enhanced survival with HSP27 expression but inverse correlation has been reported too. Inconsistent values could be due to multi-factorial oncogenesis [2,22]. Several proteomic studies identified HSP27 as a highly overexpressed protein in OSCC. Proteomic study done by Wang et al. [2] also demonstrated that higher HSP27 expression is associated with tongue SCC invasion and metastasis.

Reduced expression of HSP27 was reported as a negative prognostic factor in oesophageal carcinoma but overexpression was related poor diagnosis in astrocytoma [4]. Ito et al. [15] reported that HSP27 did not have any correlation with staging, histological grade, survival and metastatic potential. Mese et al. [23] reported reduced HSP27 expression in PDSCC than WDSCC but did not have any interrelation with clinical staging. Zhu et al. [24] observed HSP27 overexpression in a head and neck squamous cell carcinoma from a metastatic lymph node, but it was rarely expressed in the primary cancer cells from the same patient. HSP27 exists as large oligomers that reorganize to dimers and tetramers after phosphorylation. Increased HSP levels in tumor cells could be attributed to increased expression of HER & c-Myc and loss of p53 function. HSP27 enhance tumor cell growth and promote tumor cell survival by inhibition of caspase-dependent apoptosis. HSP27 has a dual role that can impart resistance of chemotherapy and can also induce CD8+ T cell-mediated anti-tumor immune responses if released into circulation [25].

Wei et al. performed a meta-analysis of HSP expression in oral cancer [22]. The pooled hazards ratio revealed that HSP27 protein overexpression significantly increased the survival rate of oral cancer patients. HSP27 might have different roles in different subtypes of oral cancers, and tumor stage might affect the HSP27 expression.

Little progress has been made in raising survival rates over the previous 30 years, despite numerous advancements in treatment. Therefore, preventing it and developing technology that makes early identification of OSCC easier that could increase survival and quality of life [1]. There is a need for extensive studies with large sample size and detailed clinical information, especially clinico-pathological data to observe the effects of HSP family proteins in various parameters of oral cancer including risk, progression, and prognosis [22-29].

Tekkesin et al. [3] reported higher HSP27 expression in leukoplakia and OSCC than in normal mucosa but no difference in HSP27 expression was apparent between OSCC and leukoplakic lesions. Similarly, we have observed significant increase in HSP27 expression from ED and OSCC but did not observe any significant difference in HSP27 levels between these two groups.

Limitations

Our study had a smaller sample size among the study groups. Other HSP family proteins like HSP70 and HSP90 could be studied. A multi-centric study with detailed clinico-pathological correlation is needed to ascertain the actual role of HSP27 expression among various grades of dysplasia and subtypes of OSCC. Once the HSP27 expression is validated, it could be used to devise a prognostic model in the future. Variations in HSP27 levels at the time of diagnosis and post-radiotherapy or post-chemotherapy will add valuable information in establishing a treatment protocol for patients with OSCC.

## Conclusions

In cases of OSCC, expression of HSP27 should be investigated more thoroughly using larger sample sizes of OSCC with a complete record of etiological factors and clinical findings. Detailed immunohistochemical analysis of HSP27 expression could highlight its role in ED and OSCC. HSP27 would definitely prove to be a good prognostic marker and help us in defining treatment modalities for OSCC, so that mortality and morbidity associated with OSCC could be reduced.

## References

[1] Deyhimi P, Azmoudeh F (2012). HSP27 and HSP70 expression in squamous cell carcinoma: an immunohistochemical study. Dent Res J (Isfahan).

[2] Wang A, Liu X, Sheng S (2009). Dysregulation of heat shock protein 27 expression in oral tongue squamous cell carcinoma. BMC Cancer.

[3] Tekkesin MS, Mutlu S, Aksakalli N (2011). Expression of heat shock proteins 27, 60 and 70 in oral carcinogenesis: an immunohistochemical study. Turk J Oncol.

[4] Lo Muzio L, Campisi G, Farina A (2006). Prognostic value of HSP27 in head and neck squamous cell carcinoma: a retrospective analysis of 57 tumours. Anticancer Res.

[5] Kaur J, Srivastava A, Ralhan R (1996). p53-HSP70 complexes in oral dysplasia and cancer: potential prognostic implications. Eur J Cancer B Oral Oncol.

[6] Mosser DD, Caron AW, Bourget L, Denis-Larose C, Massie B (1997). Role of the human heat shock protein hsp70 in protection against stress-induced apoptosis. Mol Cell Biol.

[7] Volm M, Koomägi R, Mattern J (1995). Heat shock (hsp70) and resistance proteins in non-small cell lung carcinomas. Cancer Lett.

[8] Song J, Takeda M, Morimoto RI (2001). Bag1-Hsp70 mediates a physiological stress signalling pathway that regulates Raf-1/ERK and cell growth. Nat Cell Biol.

[9] Sugerman PB, Savage NW, Xu LJ (1995). Heat shock protein expression in oral epithelial dysplasia and squamous cell carcinoma. Eur J Cancer B Oral Oncol.

[10] Kaur J, Srivastava A, Ralhan R (1998). Expression of 70-kDa heat shock protein in oral lesions: marker of biological stress or pathogenicity. Oral Oncol.

[11] Ciocca DR, Calderwood SK (2005). Heat shock proteins in cancer: diagnostic, prognostic, predictive, and treatment implications. Cell Stress Chaperones.

[12] Lo WY, Tsai MH, Tsai Y (2007). Identification of over-expressed proteins in oral squamous cell carcinoma (OSCC) patients by clinical proteomic analysis. Clin Chim Acta.

[13] Suvarna KS, Layton C, Bancroft JD (2018). Bancroft's Theory and Practice of Histological Techniques, 8th Edition. Bancroft. Bancroft's Theory and Practice of Histological Techniques, 8th Edition.

[14] Seoane JM, Varela-Centelles PI, Ramirez JR, Cameselle-Teijeiro J, Romero MA, Aguirre JM (2006). Heat shock proteins (HSP70 and HSP27) as markers of epithelial dysplasia in oral leukoplakia. Am J Dermatopathol.

[15] Ito T, Kawabe R, Kurasono Y, Hara M, Kitamura H, Fujita K, Kanisawa M (1998). Expression of heat shock proteins in squamous cell carcinoma of the tongue: an immunohistochemical study. J Oral Pathol Med.

[16] Leonardi R, Pannone G, Magro G, Kudo Y, Takata T, Lo Muzio L (2002). Differential expression of heat shock protein 27 in normal oral mucosa, oral epithelial dysplasia and squamous cell carcinoma. Oncol Rep.

[17] Parcellier A, Gurbuxani S, Schmitt E (2003). Heat shock proteins, cellular chaperones that modulate mitochondrial cell death pathways. Biochem Biophys Res Commun.

[18] Bramanti TE, Dekker NP, LozadaNur F (1995). Regezi JA.Heat Shock (stress) proteins and γδ T lymphocytes in oral lichen planus. Oral Surg Oral Med Oral Pathol Oral Radiol Endod.

[19] Tyagi N, Shetty DC, Urs AB (2012). Altered expression of HSP70 in oral lichen planus. J Oral Maxillofac Pathol.

[20] Nakajima M, Kuwano H, Miyazaki T (2002). Significant correlation between expression of heat shock proteins 27, 70 and lymphocyte infiltration in esophageal squamous cell carcinoma. World J Gastroenterol.

[21] Lo Muzio L, Leonardi R, Mariggiò MA (2004). HSP 27 as possible prognostic factor in patients with oral squamous cell carcinoma. Histol Histopathol.

[22] Lu W, Wang Y, Gan M, Duan Q (2021). Prognosis and predictive value of heat-shock proteins expression in oral cancer: a PRISMA-compliant meta-analysis. Medicine (Baltimore).

[23] Mese H, Sasaki A, Nakayama S, Yoshioka N, Yoshihama Y, Kishimoto K, Matsumura T (2002). Prognostic significance of heat shock protein 27 (HSP27) in patients with oral squamous cell carcinoma. Oncol Rep.

[24] Zhu Z, Xu X, Yu Y, Graham M, Prince ME, Carey TE, Sun D (2010). Silencing heat shock protein 27 decreases metastatic behavior of human head and neck squamous cell cancer cells in vitro. Mol Pharm.

[25] Vidyasagar A, Wilson NA, Djamali A (2012). Heat shock protein 27 (HSP27): biomarker of disease and therapeutic target. Fibrogenesis Tissue Repair.

[26] Wang Y, Zhang Y, Guo Y (2019). Synthesis of Zinc oxide nanoparticles from Marsdenia tenacissima inhibits the cell proliferation and induces apoptosis in laryngeal cancer cells (Hep-2). J Photochem Photobiol B.

[27] Rohit Singh T, Ezhilarasan D (2020). Ethanolic extract of Lagerstroemia speciosa (L.) Pers., induces apoptosis and cell cycle arrest in HepG2 cells. Nutr Cancer.

[28] Subramaniam N, Muthukrishnan A (2019). Oral mucositis and microbial colonization in oral cancer patients undergoing radiotherapy and chemotherapy: a prospective analysis in a tertiary care dental hospital. J Investig Clin Dent.

[29] Wadhwa R, Paudel KR, Chin LH (2021). Anti-inflammatory and anticancer activities of naringenin-loaded liquid crystalline nanoparticles in vitro. J Food Biochem.

